# Capillary condensation-driven growth of perovskite nanowire arrays for multi-functional photodetector

**DOI:** 10.1038/s41377-024-01680-2

**Published:** 2025-01-24

**Authors:** Gangjian Hu, Jiajun Guo, Jizhong Jiang, Lei Wang, Jiaqi Zhang, Hongxu Chen, Gangning Lou, Wei Wei, Liang Shen

**Affiliations:** 1 State Key Laboratory of Integrated Optoelectronics, College of Electronic Science and Engineering, International Center of Future Science, Jilin University, Changchun, 130012 China; 2 Westlake Institute for Optoelectronics, Fuyang, Hangzhou 311421 China; 3 College of Materials Science and Engineering, Key Laboratory of Automobile Materials, Ministry of Education, Jilin University, Changchun, 130012 China

**Keywords:** Nanowires, Imaging and sensing

## Abstract

Metal-halide perovskite nanowire array photodetectors based on the solution method are valuable in the field of polarized light detection because of their unique one-dimensional array structure and excellent photoelectric performance. However, the limited wettability of liquids poses challenges for achieving large-scale and high-quality perovskite nanowire arrays. To address this issue, we develop a facile method utilizing capillary condensation to grow high-quality centimeter-scale perovskite nanowire arrays. Based on these nanowires, the fabricated photodetector showcases specific detectivities of 1.95 × 10^13^ jones, surpassing commercially available silicon detectors in weak-light detection capabilities. The weak-light imaging capability of our nanowire photodetectors has been successfully demonstrated at intensities below 54 nW/cm^2^. Moreover, the nanowire arrays also display excellent polarization absorption characteristics, promising applications in polarized light detection. Notably, the perovskite nanowire arrays can be grown on flexible substrates by employing capillary condensation, which retains 83% of their properties after 2000 bending cycles. This research enhances the potential of perovskite nanowire arrays photodetector in practical applications.

## Graphical abstract

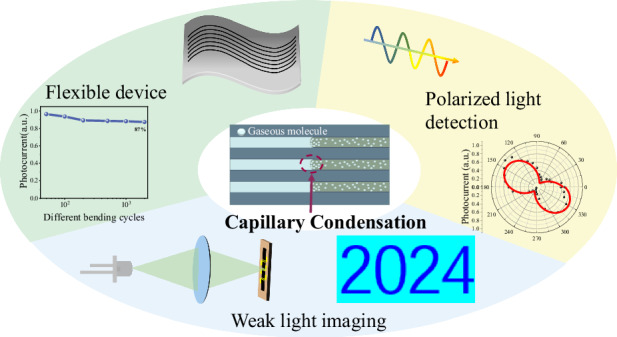

## Introduction

The organic and inorganic hybrid perovskite materials, being a novel class of photoelectric materials, exhibit a high optical absorption coefficient, long carrier diffusion length, tunable bandgap, facile preparation process, etc., rendering them highly promising for applications in solar cells, light-emitting diodes and photodetectors^1–12^. Compared to three-dimensional and two-dimensional materials, one-dimensional nanowire materials exhibit a significantly enhanced surface area-to-volume ratio, an abundance of surface states, and superior bending resistance. Consequently, they possess greater potential for applications in weak-light detection, flexible devices, and polarized light detection^13–16^. Therefore, synthesizing high-quality perovskite nanowire arrays (NAs) has become a focal point for the academic community, with numerous important advancements made in relevant research.

Significant advancements have been achieved in the investigation of template-assisted growth for fabricating highly ordered perovskite NA photodetectors. The capillary forces were employed by Wu et al. to facilitate the growth of two-dimensional perovskite NAs on silicon substrates, resulting in a specific detectives value of 7 × 10^15^ jones, which undeniably demonstrates the promising application potential of perovskite nanowire photodetectors^17^. However, the growth process is relatively complex and needs selective modification of the template. According to research findings, capillary-driven infiltration behavior at the nanoscale only enables the formation of liquid columns with lengths in the range of hundreds of microns, which satisfies the fabrication requirements for a limited number of devices but imposes constraints on the potential applications of perovskite NAs^18^. The MAPbBr_3_ microwire-array photodetectors were fabricated by Li et al. using micron-scale capillaries, exhibiting a specific detectivity value of 4.1 × 10^11^ jones. Despite the expansion of capillary size to the micron scale, the length of the grown microwires only reaches the millimeter scale^19^. Deng et al. fabricated large-area MAPbI_3_ microwire-array photodetectors with a remarkable detectivity of 5.25 × 10^12^ jones through the utilization of a blade coating technique^20^. However, achieving a highly ordered arrangement of microwave arrays prepared by this method remains challenging, thereby limiting its potential for polarization detection and precluding the fabrication of nanoscale arrays.

In this contribution, we employed capillary condensation to grow high-quality and large-scale NAs of MAPbI_3_ and MAPbBr_3_ perovskite. Capillary condensation is a phenomenon wherein gaseous molecules undergo the process of condensation into liquid within a capillary, and this occurrence is further intensified as the size of the capillary decreases^21^. Consequently, the issue of hindered solution movement in nanoscale capillaries can be effectively addressed by harnessing the phenomenon of capillary condensation, thereby facilitating substantial filling of the capillaries between the substrate and the PDMS template. The capillary condensation method for preparing perovskite NAs not only eliminates the need for substrate and template processing but also offers a facile growth process that enables large-scale continuous production of perovskite NAs. Photodetectors based on this preparation method exhibit exceptional photoelectric detection performance and hold great potential in areas such as polarization detection, flexible devices, and low-light detection.

## Results

**Fig. 1:**
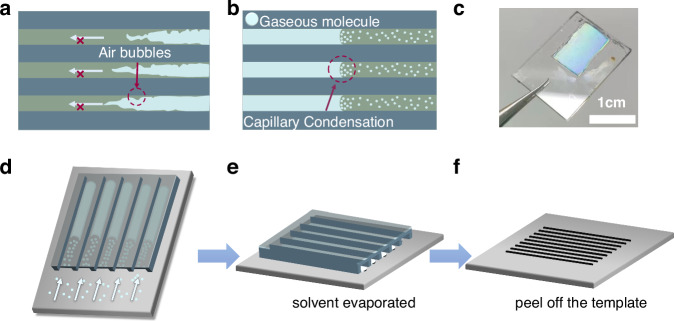
**Nanowire growth process by capillary condensation. a** capillary filling process in nanochannel; **b** capillary condensation process in nanochannel; **c** MAPbI_3_ NA image; **d** diagram of infiltration of capillary channels by capillary condensation; **e** the substrate is placed on a heated table set at a specific temperature to conduct an annealing process; **f** peel off the PDMS template, and the nanowires are arranged periodically on the substrate

An important process for growing NAs using the template method is that the precursor solution fills the capillary tubes formed between the template and the substrate. The current method uses capillary force to drive this process, and the pressure, *P*, for driving the capillary filling process can be given in the following equation:

1 $$P=\frac{2\gamma \cos {\theta }_{E}}{R}$$

where *γ* is the surface tension of liquid, *θ*_*E*_ the contact angle, *R* the radius of capillary tube. It can be known from the equation that the capillary force driving the liquid flow is closely related to the size of the contact angle of the material. Therefore, there are certain requirements for the template and the substrate material, and certain treatment is required for the template. The Kelvin equation predicts the humidity required for capillary condensation to occur^20^:

2 $${{RH}}_{K}=\exp \left(\frac{-2\gamma }{{k}_{B}{TR}{\rho }_{N}}\right)$$

where RH is the humidity, T is the temperature, *k*_*B*_ is the Boltzmann constant, and *ρ*_*N*_ is the number density of liquid. From the equation, it can be observed that the phenomenon of capillary condensation primarily depends on the size of the capillary tube and the density of the solution itself, and is not influenced by the contact angle of the material. Consequently, the selectivity of the substrate material is broadened, and the preparation process is simplified. Additionally, compared to capillarity-driven filling in nanochannels, which can be easily obstructed by air bubbles, capillary condensation exploits the motion of gaseous molecules to replace liquid flow within nanochannels, as illustrated in Fig. 1a, b. Utilizing the capillary condensation phenomenon, we devised a methodology for the fabrication of perovskite NAs, as depicted in Fig. 1d, e. The PDMS templates depicted in Fig. 1 were fabricated using compact discs, and detailed preparation steps can be found in the experimental section. The PDMS template with nanostructures is firmly affixed to the glass substrate, establishing a periodic capillary interface between them. Subsequently, the substrate is immersed in a hermetically sealed quartz crucible filled with perovskite precursor solution. To accelerate the condensation of the liquid within the capillary, controlled heating was applied to the quartz crucible on a hot plate, thereby facilitating the evaporation of the perovskite precursor solution. After undergoing the heating treatment, the capillary is filled with the precursor solution. Subsequently, the substrate is extracted and placed onto a hot plate set at a specific temperature for annealing treatment. Following annealing, the PDMS template is carefully peeled off to yield a well-ordered array of perovskite nanowires. Due to the small size of the capillary and the absence of any hydrophilic/hydrophobic treatment, direct infiltration of perovskite precursor solution into the capillary via capillary force was hindered, or only a partial filling could be achieved (Fig. S1). Conversely, employing the capillary condensation method enabled the gradual and complete filling of the entire capillary with perovskite precursor solution, as illustrated in Fig. 1c. The morphology of the prepared nanowires primarily depends on the shape of the channel used in the template. The width of the prepared NA primarily depends on the width of the template channel used. Since we use templates of uniform size, the widths of all the nanowires are essentially the same. The thickness of the nanowires is influenced not only by the height of the template channel but also by the concentration of the precursor solution. Higher concentrations result in thicker perovskite nanowires. The length of the nanowires is mainly determined by the length of the capillary liquid column formed. Heating is employed to enhance the concentration of gaseous molecules within the capillary. By precisely controlling both temperature and duration, we can regulate the rate and total amount of gaseous molecules condensing into liquid, thereby controlling the length of the prepared nanowires. However, achieving perovskite NAs solely through capillary condensation is time-consuming in the actual growth processs. Therefore, we usually immerse one end of the nanochannel in the solution and exploit both capillary force and capillary condensation to expedite solution infiltration.

**Fig. 2:**
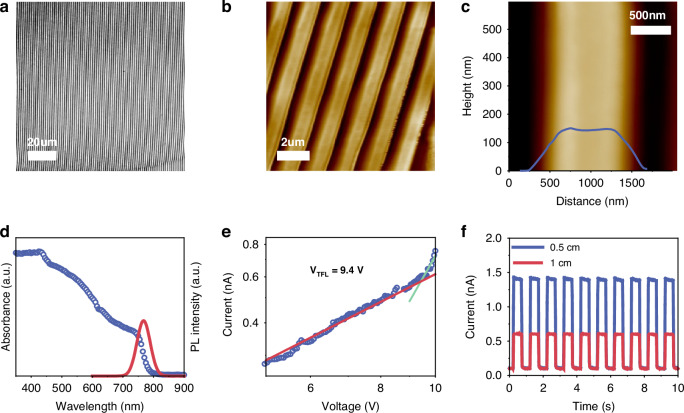
**Characterization** **of** **MAPbI**_**3**_ **NAs.** **a** NA images under the optical microscope; **b**, **c** AFM image of MAPbI_3_ NA; **d** absorption and photoluminescence spectrum of MAPbI_3_ NA, where the inset shows the corresponding Tauc plots to extract the bandgap; **e** I–V trace of the MAPbI_3_ NA; **f** the optical response test of large-area MAPbI_3_ nanowire devices was conducted with an electrode spacing of 0.5 cm and 1 cm

The MAPbI_3_ NA prepared by capillary condensation is not only uniform on the macro-scale but also uniform on the micro-scale. The optical image of the NA under a 100-fold optical microscope is presented in Fig. 2a. It can be observed from the figure that the grown nanowires exhibit a significant area of continuity and excellent uniformity at the scale of hundreds of microns. The morphology of the grown nanowires was further examined using atomic force microscopy (AFM) and scanning electron microscopy (SEM), as illustrated in Figs. 2b, c and S2. The absence of discernible grain boundaries, both under AFM and SEM observation, attests to the exceptional uniformly and single-crystal properties exhibited by our fabricated NAs across macroscopic and microscopic dimensions. Furthermore, under high-resolution SEM observation, the individual nanowires exhibited clear edges and flat surfaces, with no obvious dents or burrs. This indicates that morphological defects in the nanowires were relatively minimal, consistent with our previous analysis which suggested that the defects were primarily due to unpaired dangling bonds on the surface. The absorption spectrum of MAPbI_3_ NA, as shown in Fig. 2d, exhibits a distinct band edge cutoff without any excitonic signature. By analyzing the Tauc plot, the optical bandgap of MAPbI_3_ NA can be determined to be 1.57 eV. We also conducted photoluminescence (PL) spectroscopy on MAPbI_3_ NA, revealing a PL peak at 767 nm with a full width at a half-maximum of ~48 nm. To determine the trap density of MAPbI_3_ NA, we employed the space-charge-limited current (SCLC) method (Fig. 2e). The SCLC test was conducted in a dark environment. According to SCLC theory, the trap density (*n*_t_) can be calculated by the Eq. (3)

3 $${n}_{t}=\frac{2{V}_{{TFL}}\varepsilon {\varepsilon }_{0}}{e{L}^{2}}$$

where *V*_TFL_ is the trap-filled limit voltage, *ε* is the relative dielectric constant, *ε*_0_ is the vacuum permittivity, and *L* is the length of the nanowires between the two electrodes^22^. The defect state density, n_t_, was calculated to be 6.7 × 10^13 ^cm^−3^. Compared to perovskite films, the defect state density of MAPbI_3_ NAs is significantly reduced by 3 orders of magnitude. The obtained value is in accordance with the high-quality NAs fabricated using the capillary-bridge method, thereby validating the superior quality of the NAs produced via capillary condensation^13,19^. Although the defect state density still surpasses that of certain perovskite single crystals, we posit that the defects in the grown nanowire primarily originate from chemically bonded species suspended on its surface. Furthermore, these surface defects serve as one of the sources for the photoconductive gain observed in nanowire materials under weak-light irradiation, thereby enhancing device performance^23^. Additionally, we successfully demonstrated the centimeter-scale continuity of the prepared nanowires by evaporating transverse electrodes 1 cm apart on the NA and assessing its photoelectric response under a bias voltage of 5 V, and a monochromatic light source emitting at 365 nm with an intensity of 382 μW/cm^2^, as illustrated in Fig. 2f. The successful synthesis of high-quality single-crystalline and large-area continuous MAPbI_3_ NA was confirmed through the combination of these characterizations using our capillary condensation method.

**Fig. 3:**
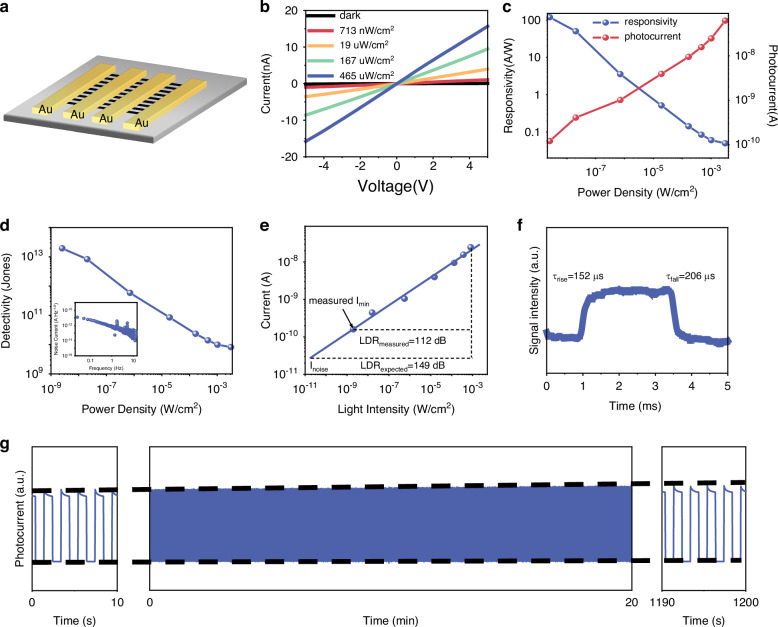
**Performance characterization of MAPbI**_**3**_ **NA photodetectors.** **a** schematic diagram of the photodetector’s structure; **b** I–V curve of the photodetector in a dark state and under 365 nm light irradiation; **c** the photogenerated current and the corresponding responsivity of the device under different light intensity 365 nm light irradiation at a bias voltage of 5 V; **d** the dependence of the detectivity of the device on the intensity, at a bias voltage of 5 V, insert: noise power density spectra of the device at 5 V bias; **e** the dependence of the current of the device on the light intensity, at a bias voltage of 5 V; **f** response speed of the device; **g** operational stability test of the photodetector

To further investigate the photoelectric properties of the prepared MAPbI_3_ NA, we fabricated a device with the structure illustrated in Fig. 3a. The transverse gold electrode was fabricated on the surface of the NA using vacuum evaporation. The evaporation rate was ~0.02 nm/s. The size and spacing of the electrodes were precisely controlled by a mask. The distance between the gold electrodes measures 20 um, while the width is 2 mm. Consequently, the area of an individual device amounts to 4 × 10^−4^ cm^2^. Initially, we conducted I–V curve measurements on the device both in darkness and under varying light intensities at a wavelength of 365 nm as depicted in Fig. 3b and the photodetector exhibited ohmic contact characteristics. All measurements of the device were conducted at a temperature of 25 °C, under normal atmospheric pressure, and at 20% humidity. The responsivity of the photodetector can be defined by

4 $$R=\frac{I}{P}$$

where *I* is the photocurrent and *P* is the input power of light source^24–28^. The variation of photocurrent and responsivity with input power density under a 5 V bias is shown in Fig. 3c. According to Eq. (4), the responsivity of the device at 5 V is 118 A/W. The photodetector’s ability to detect weak signals can be quantified by the specific detectivity (D*), which is determined by the responsivity and noise characteristics of the photodetectors. This parameter can be calculated using the following equations.

5 $${D}^{* }=\frac{\sqrt{{AB}}}{{NEP}}\left({cm}{{Hz}}^{-1/2}{W}^{-1}{or\; jones}\right)$$

6 $${NEP}=\frac{{i}_{n}}{R}\left(W\,{{Hz}}^{-\frac{1}{2}}\right)$$

where A is the active layer area, *i*_*n*_ is the noise current, B is the bandwidth and NEP is the noise equivalent power^29–31^. The dark current is as low as 3.91 × 10^−11^ A at 5 V, indicating a relatively low noise current and high sensitivity. As shown in Fig. 3d (inset), we selected the mean value of 1.21 × 10^−13^ A Hz^−1/2^ near the 10 Hz point as the device’s noise current. This value is approximately an order of magnitude lower than that of the MAPbI_3_ thin film photodetector^3^. We attribute the low noise current to the reduced defect state density within the prepared perovskite nanowires and the suppression of noise current by the one-dimensional structure of the nanowires. The D* is calculated as 1.95 × 10^−13^ jones, indicating the high performance of the fabricated nanowire photodetector. We compared the performance of the prepared NA photodetector with other perovskite nanowire/ microwire photodetectors (Table S1) and demonstrated the advantage of the capillary condensation mechanism in preparing high-performance nanowire detectors. We prepared 64 devices and measured their responsivity at 50 nW/cm². The results, shown in Figure S3, indicate that most devices exhibit excellent performance, while a few show poor performance. We believe this variability is mainly due to the uneven fit between the PDMS template and the substrate. The linear light response range is always characterized by the linear dynamic range (LDR), which refers to the optical power range which the output photocurrent exhibits a linear relationship with the input optical signal, and can be calculated from the Eq. (5):

7 $${LDR}=20\times \log \frac{{P}_{\max }}{{P}_{\min }}$$

where *P*_max_ and *P*_min_ are the upper and lower limits of the optical power^32–35^. A sufficiently large LDR ensures that the device maintains a linear photocurrent response across varying light intensities, from strong to weak illumination, which is crucial for weak-light sensing. As shown in Fig. 3e, the LDR of the photodetectors under 365 nm LED illumination reveals a linear photocurrent increase across a dynamic light intensity range from 2 nW/cm^2^ to 0.8 mW/cm^2^, corresponding to an LDR value of 112 dB. To achieve a more accurate assessment of the actual LDR, we employed the noise current derived from the noise power density spectra, yielding an estimated LDR value of 149 dB. This value is on par with that of inorganic semiconductor detectors^16^. The observed linear response can be attributed to the excellent carrier transport properties and low electron trap density in the photodetector. The rising and falling times are crucial parameters for characterizing the response speed of the photodetector^36,37^. The response speed characteristics of the device to pulsed light is evaluated under a bias voltage of 5 V. As depicted in Fig. 3f, the device exhibited a rise time of 152 µs and a fall time of 206 µs, thereby demonstrating its remarkable fast response speed. The operational stability of the device is a crucial parameter for evaluating its potential applications^38^. The device operates continuously for a duration of 20 minutes, subjected to a 5 V bias and continuous irradiation from a pulsed light source. The oscilloscope generates a pulse voltage to drive the 365 nm LED, thereby producing pulsed light. The corresponding test results are illustrated in Fig. 3g. The results demonstrate the detector’s exceptional stability without any signal attenuation, thereby confirming its robust operational stability. The unique anisotropic crystalline properties of MAPbI_3_ make it a highly suitable material for the growth of perovskite nanowires^39^. Due to the different crystallization modes of MAPbBr_3_ and MAPbI_3_ materials, methods such as blade coating and low-temperature crystallization can only prepare MAPbI_3_ nanowires. To validate the versatility of our approach in fabricating perovskite nanowires with varying compositions, we also fabricated photodetectors based on MAPbBr_3_ nanowires, with the relevant characterization presented in Figure S4. The responsivity of the fabricated devices reached 10 A/W, while exhibiting a D* of 3.43 × 10^12^ jones. This performance demonstrates the applicability of our methodology for fabricating perovskite NAs across various material systems.

**Fig. 4:**
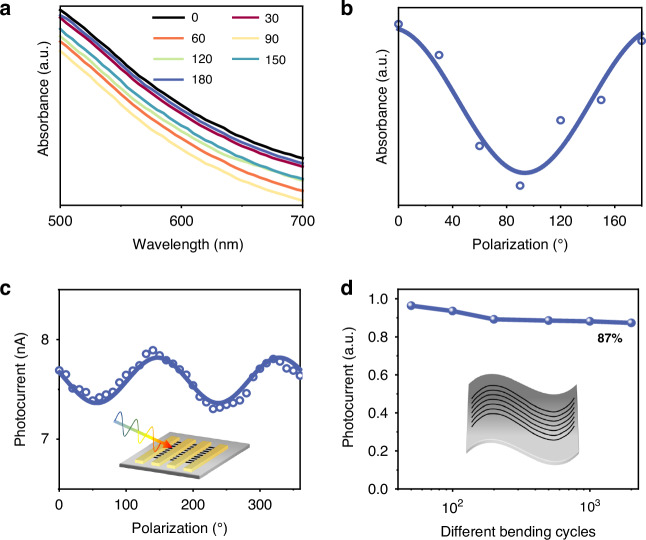
**Polarization and flexible characterization of MAPbI**_**3**_ **NA.** **a** absorption spectra of MAPbI_3_ NA for polarized light at various angles; **b** the relationship between the absorption coefficient of 600 nm light and the polarization angle; **c** polarized light response characteristics of MAPbI_3_ NA photodetector; **d** normalized photocurrent before and after different bending cycles at a fixed bending radius (6 mm)

The anisotropic structure and exceptional photoelectric properties of the MAPbI_3_ NA render it a promising candidate for polarization-sensitive photodetectors^40,41^. The absorption spectrum of MAPbI_3_ NA was investigated using polarized light at different angles. It is observed that the absorption coefficient shows periodic variations with changes in polarization angle, as shown in Fig. 4a. To visually demonstrate this variation within the 600 nm wavelength range, we plotted the correlation between the absorption coefficient and polarization angle, as depicted in Fig. 4b, revealing distinct characteristics of polarization absorption. Furthermore, we conducted an assessment of the response characteristics of the fabricated device toward polarized light at various angles. The polarization detection system employs a 600 nm LED light source in conjunction with a linear polarizer. As illustrated in Fig. 4c, the photocurrent variation of the device adheres to a sinusoidal function, thereby substantiating its capability for polarized light detection. The unique structural characteristics of nanowires endow them with significant potential for applications in flexible electronics^42,43^. Consequently, we have successfully fabricated MAPbI_3_ NAs on flexible PEN substrates utilizing capillary condensation. We characterized the performance of flexible MAPbI_3_ NA (Fig. S5) and analyzed its photocurrent under bending conditions. Flexible MAPbI_3_ NA also exhibits polarization absorption characteristics, as shown in Fig. S6. Figure 4d describes the change in photocurrent of the flexible device with different bending cycles. The photocurrent of the device exhibited a negligible decrease as the number of bending cycles increased. Even after undergoing 2000 cycles, the device maintained 87% of its initial response, thereby demonstrating exceptional durability. The aforementioned experimental results demonstrate the application potential of our detector in the field of flexible and polarized light detection.

**Fig. 5:**
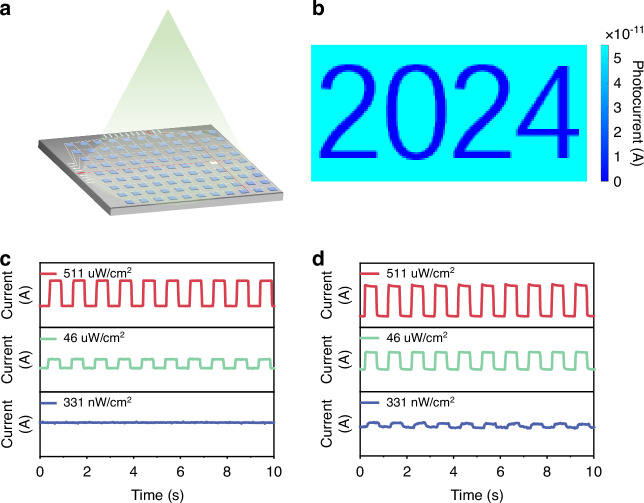
**Weak light detection performance of MAPbI**_**3**_ **NA photodetector.** **a** the detection mechanism of the area array photodetector; **b** imaging result of MAPbI_3_ NA photodetector; **c** commercial silicon detector light response at 365 nm; **d** MAPbI_3_ NA photodetector light response at 365 nm

Weak-light detection holds significant application value in the fields of imaging, biomedicine, and other fields. To ensure high-resolution imaging, it is crucial to minimize the area of individual detectors on the imaging chip, which leads to a reduced number of photons received by each detector in low-light conditions, as depicted in Fig. 5a. Consequently, this places stringent demands on the photoelectric performance of the photodetector. To assess the potential of NAs photodetectors for weak-light detection, we analyze the response between commercial silicon photodetectors and MAPbI_3_ NAs photodetectors under pulsed light conditions. To ensure the same number of photons reception by both detectors at identical optical power densities, a partial light transmission template is integrated into the silicon detector surface. This design makes the photosensitive area of both detectors to 0.04 mm². The performance of the commercial silicon detector is shown in Fig. S7. As depicted in Fig. 5c, the silicon detector exhibits negligible light response when subjected to 365 nm light irradiation at an intensity of 331 nW/cm^2^. However, our NAs photodetector exhibits a distinct photoelectric response under the light irradiation of 331 nW/cm^2^ and maintains its resolved response when the incident light power density is reduced to 14.5 nW/cm^2^, as illustrated in Figs. 5d and S8. According to the absorption curve in Fig. 2e, the MAPbI_3_ NA exhibits a significantly high absorption coefficient for light at 365 nm, while the response of silicon photodetectors to UV light is relatively weak compared to other spectral bands. Therefore, we also conducted a photoelectric response comparison test using 660 nm light, as shown in Fig. S8. Our NA photodetector continues to outperform commercial silicon photodetectors. This result demonstrates that the low-light detection capability of our NA photodetector not only surpasses that of commercial silicon detectors in the ultraviolet band but also in the visible band, where the absorption coefficient is relatively weak. This highlights the exceptional low-light detection capability of our NA photodetector across a wide spectrum. We constructed an imaging system, as depicted in Fig. 5b, to evaluate the imaging capability of the prepared MAPbI_3_ NA photodetector under weak-light conditions. Throughout the entire imaging process, the photodetector was exposed to monochromatic light with a wavelength of 365 nm, while the optical power density did not exceed 54 nW/cm^2^. Thanks to the exceptional weak-light detection capability of MAPbI_3_ NA photodetector, we successfully obtained a clear image of “2024” under such weak illumination conditions.

## Discussion

In summary, we have developed a facile approach for the growth of large-area, high-quality perovskite NAs via capillary condensation. Benefiting from the exceptional nanowire quality, the MAPbI_3_ NA exhibits an impressive responsivity of 118 A/W and a D* of 1.95 × 10^13^ jones, while the MAPbBr_3_ counterpart demonstrates a responsivity of 10 A/W and a D* of 3.43 × 10^12^ jones. Moreover, besides their outstanding photoelectric properties, these grown NAs also hold great potential in applications such as polarization detection and flexible devices. By evaluating both the polarization absorption characteristics of the MAPbI_3_ NA and the light response characteristics of our device, we have demonstrated its capability for polarized light detection. Furthermore, we successfully employed capillary condensation to grow MAPbI_3_ NAs on a flexible PEN substrate and fabricated corresponding devices. Even after undergoing 2000 bending cycles, the flexible device performance remained at 87% with excellent imaging capabilities under bending conditions, highlighting our nanowire photodetectors’ immense potential in the flexible detectors field. We also conducted a comparative analysis of the weak-light detection capabilities between commercial silicon detectors and nanowire photodetectors, both for ultraviolet and visible light. Notably, the NA photodetectors have better performance in both light. We anticipate that this work will provide a pathway for the development of multi-functional perovskite NA photodetectors, facilitating their future commercialization.

## Materials and methods

### Material

lead bromide (PbBr_2_) (>98%, Xi’an Polymer Light Technology Corp), lead iodide (PbI_2_) (>98%, Xi’an Polymer Light Technology Corp), methylamine bromide (>98%, Xi’an Polymer Light Technology Corp), methylamine iodide (>98%, Xi’an Polymer Light Technology Corp), DMF(>99.8%, Sigma Aldrich). All chemicals were used as received, without any further purification.

### Preparation of PDMS template

We obtained the PDMS template for nanowire growth by transferring the periodic structure of a CD. Firstly, the CD was trimmed to an appropriate size, and then its protective layer was removed before undergoing a 15-minute ultrasonic cleaning in anhydrous ethanol followed by another 15-minute ultrasonic cleaning in purified water. Finally, any residual surface moisture was eliminated using nitrogen gas. The PDMS prepolymer and curing agent were mixed at a weight ratio of 10:1. The mixture was placed in a negative pressure environment for 20 minutes to remove air bubbles, and subsequently spin-coated onto the processed CD template at a speed of 500 rpm for 30 seconds. After baking at 60 °C for 120 minutes, the cured PDMS film was peeled off from the CD template.

### Growth of the MAPbI_3_/MAPbBr_3_ NAs

The growth process of the NA is shown in Fig. S9 and all the processes were prepared at room temperature. 0.5 M solution of MAPbI_3_ was prepared in DMF. The solution was subjected to active mixing at 25 °C for 12 hours to ensure complete dissolution. Subsequently, the PDMS template is attached to the glass substrate and placed in a quartz crucible. The glass substrates were ultrasonically cleaned with acetone and ethanol for 20 min. 1 mL of perovskite precursor solution is added, and the quartz crucible is sealed. It is then placed on a 100 °C hot plate and heated for 1 to 12 hours, adjusting the time based on the size of the PDMS template. For a 1 cm^2^ PDMS template, the heating treatment time is 4 h. After the completion of the heating process, the perovskite precursor solution is introduced into the capillary. Subsequently, the substrate is extracted from the enclosed container and subjected to annealing on a 100 °C hot plate for 7 minutes. Finally, peel off the PDMS template and obtain MAPbI_3_ NAs. The preparation method of MAPbBr_3_ NA is identical to that of MAPbI_3_ NAs, except for a variation in annealing temperature at 80 °C.

### Perovskite NAs characterizations

SEM characterizations were conducted with the JEOL JSM-7900 field-emission scanning microscope.PL spectra were obtained from the Jobin Yvon IHR550 Imaging Spectrometer. AFM was measured by the Bruker Dimension Icon AFM in tapping mode. The UV-vis absorption characterization was conducted using the Shimadzu UV-3600 Pharma Spec UV spectrophotometer, wherein the absorption with varying polarizations was quantified by incorporating polarizers with diverse polarization angles.

### Device fabrication and measurements

The surface of NAs was coated with transverse gold electrodes, which were deposited using vacuum evaporation and had a thickness of 40 nm. The evaporation rate was approximately 0.02 nm/s. The size and spacing of the electrodes were precisely controlled by a mask. Specifically, the spacing between the transverse electrodes was set at 20 μm, while their length measured 2 mm. The I–V and noise current curve were obtained by a Keithley 4200A-SCS parameter analyzer. The noise current is tested at 5 V bias and dark state. The SCLC and noise tests were performed in a dark environment. All tests involving light will use LEDs as the light source. The LED is driven by applying either a DC or square wave voltage through an oscilloscope. The optical power density received by the device is adjusted by varying the bias amplitude and employing an external optical filter. An optical power meter is used to calibrate the actual light intensity received by the device. The rise and fall times of the device are measured by driving an LED with a square wave voltage to illuminate the device. A 5 V bias is applied to the device, and its response is monitored with an oscilloscope. The rise time is defined as the time difference between the 10% and 90% amplitude points of the rising curve, while the fall time is the time difference between the 90% and 10% points of the falling curve. The oscilloscope utilized during the experiment is the Keysight DSOX6004A, while the optical power meter employed is the Newport 843-R. The photodetector performance measurement was conducted under controlled environmental conditions, including a room temperature of 25 °C, humidity maintained at 20%, and normal atmospheric pressure.

## Supplementary information

Supplementary Information for Capillary Condensation-Driven Growth of Perovskite Nanowire Arrays for Multi-Functional Photodetector

## Data Availability

The data used in this study were provided by the corresponding author. Specific datasets can be requested from the corresponding author as needed.
