# Supplementary material for: Capillary condensation-driven growth of perovskite nanowire arrays for multi-functional photodetector
Source: Light Sci Appl. 2025 Jan 24;14:61. doi: 10.1038/s41377-024-01680-2 (PMC11761479; doi:10.1038/s41377-024-01680-2)
Supplement: Supplementary file 1 — Supplementary Information for Capillary Condensation-Driven Growth of Perovskite Nanowire Arrays for Multi-Functional Photodetector [file 41377_2024_1680_MOESM1_ESM.docx]

**Supplementary Information for Capillary Condensation-Driven Growth of Perovskite Nanowire Arrays for Multi-Functional Photodetector**

Gangjian Hu^1^, Jiajun Guo^1^, Jizhong Jiang^2^, Lei Wang^3^, Jiaqi Zhang^3*^, Hongxu Chen^1^, Gangning Lou^1^, Wei Wei^1^, Liang Shen^1,2*^

^1^State Key Laboratory of Integrated Optoelectronics, College of Electronic Science and Engineering, International Center of Future Science, Jilin University, Changchun 130012, China.

^2^Westlake Institute for Optoelectronics, Fuyang, Hangzhou 311421, China.

^3^College of Materials Science and Engineering, Key Laboratory of Automobile Materials, Ministry of Education, Jilin University, Changchun 130012, China.

KEYWORDS: capillary condensation, perovskite, nanowire arrays, photodetector

| Material | *R* (A/W) | *D** (jones) | Refs. |
| --- | --- | --- | --- |
| MAPbI_3_ nanowire | 118 | $1.95\times{10}^{13}$ | This work |
| MAPbI_3_ microwire | 13.85 | $3.87\times{10}^{12}$ | 1 |
| MAPbI_3_ nanowire | 4.95 | $2.0\times{10}^{13}$ | 2 |
| MAPbI_3_ microwire | 0.04 | $0.6\times{10}^{12}$ | 3 |
| MAPbI_3_ nanowire | 0.16 | $4.16\times{10}^{12}$ | 4 |
| MAPbI_3_ nanowire | 20.56 | $4.73\times{10}^{12}$ | 5 |
| MAPbI_3_ microwire | 13.57 | $5.25\times{10}^{12}$ | 6 |
| MAPbI_3_ microwire | 1.2 | $2.39\times{10}^{12}$ | 7 |
| MAPbI_3_ microwire | 0.48 | $1.26\times{10}^{12}$ | 8 |
| MAPbI_3_ nanowire with BMIMBF_4_ | 37.14 | $2.06\times{10}^{13}$ | 9 |

**Table S1**. Performance comparison of perovskite nanowires/microwire photodetectors reported in the literature.

**Figure S1.** Preparation of MAPbI_3_ NA was achieved via capillary force. The sequential images, from left to right, illustrate adding perovskite precursor solution to one side of the PDMS template, followed by annealing on a heated surface and subsequent removal of the PDMS template.


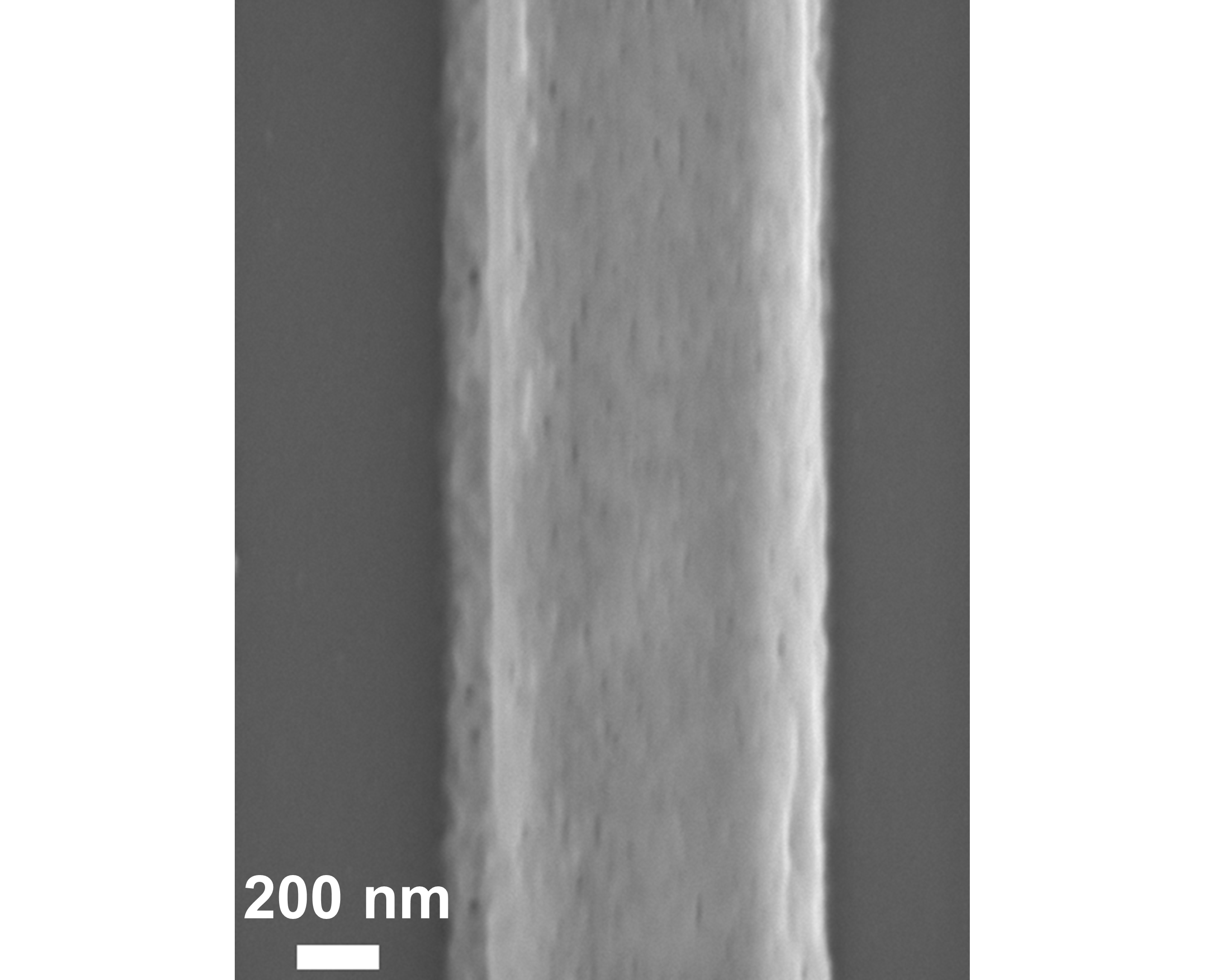


**Figure S2.** SEM image depicting a single MAPbI_3_ nanowire.


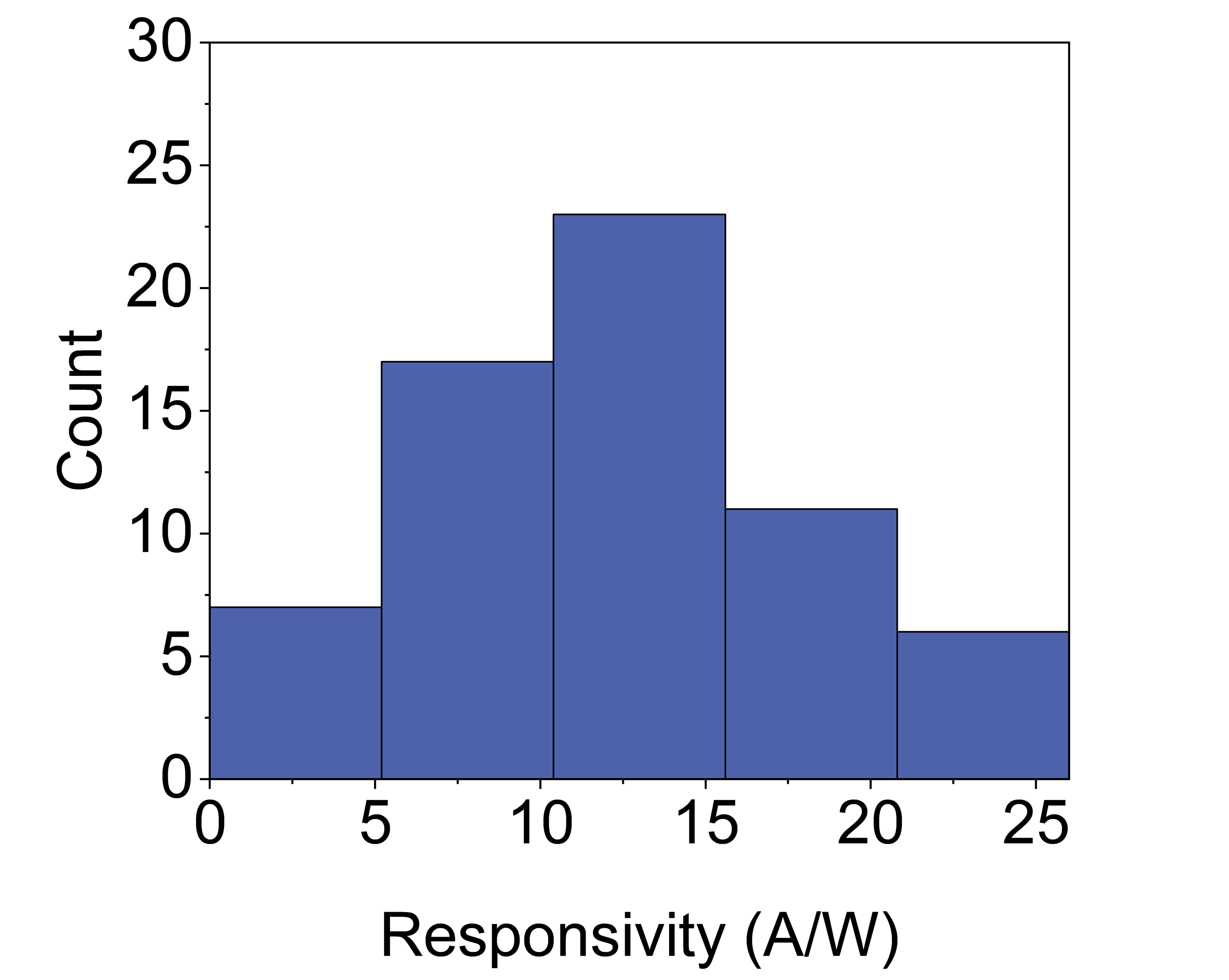


**Figure S3**. Responsivity statistics of MAPbI_3_ devices under 365 nm light at an intensity of 50 nW cm^-2^.


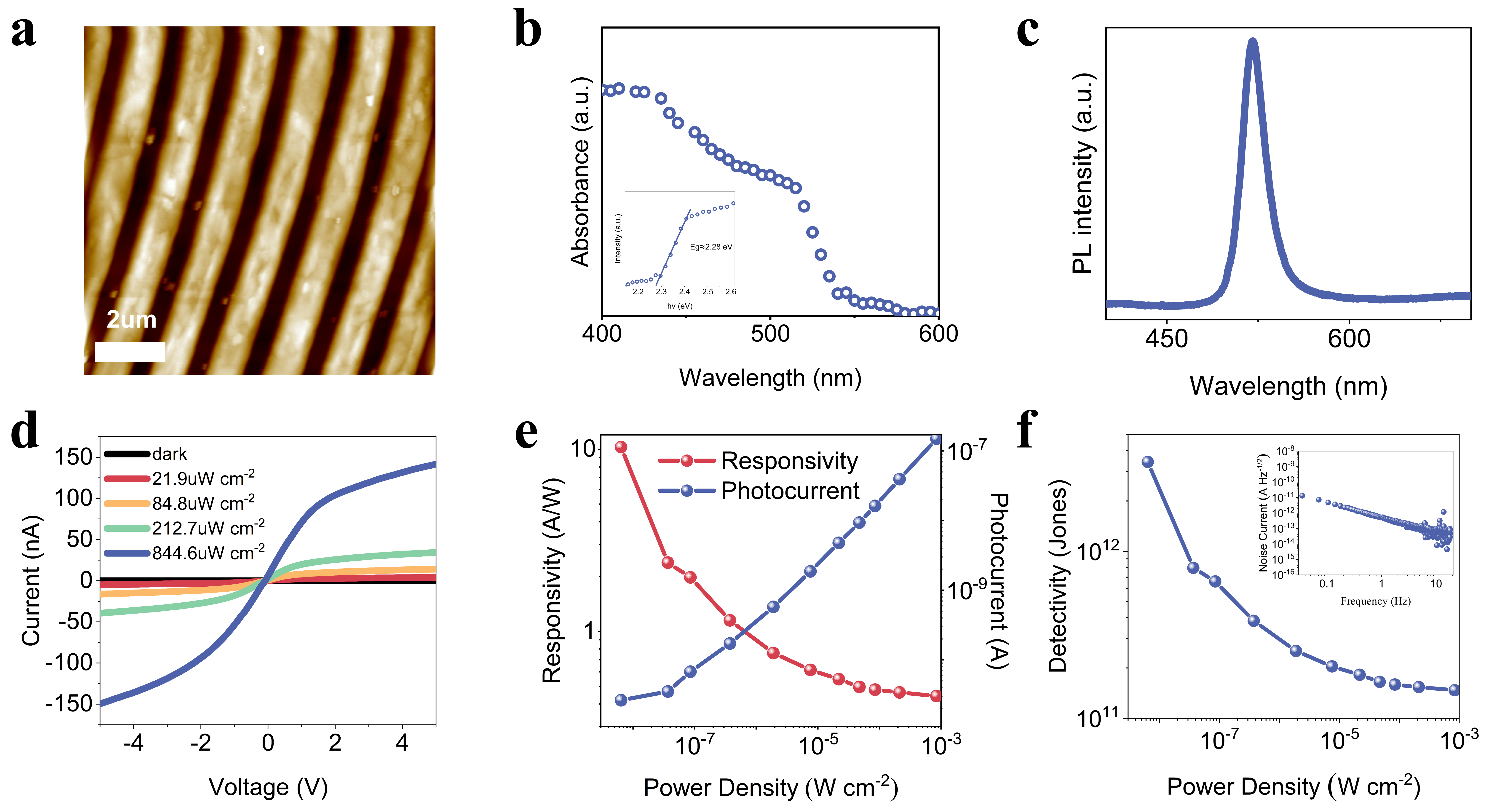


**Figure S4.** Characterization of MAPbBr_3_ NA: (a) AFM image of MAPbBr_3_ NA; (b) absorption spectrum of MAPbBr_3_ NA, where the inset shows the corresponding Tauc plots to extract the bandgap; (c) photoluminescence spectra of the MAPbBr_3_ NA; (d) I-V curve of the photodetector in the dark state and under 365 nm light irradiation; (e) the photogenerated current and the corresponding responsivity of the device under different light intensity 365 nm light irradiation at a bias voltage of 5 V; (f) the dependence of the detectivity of the device on the intensity, at a bias voltage of 5 V, insert: noise power density spectra of the device at 5 V bias.


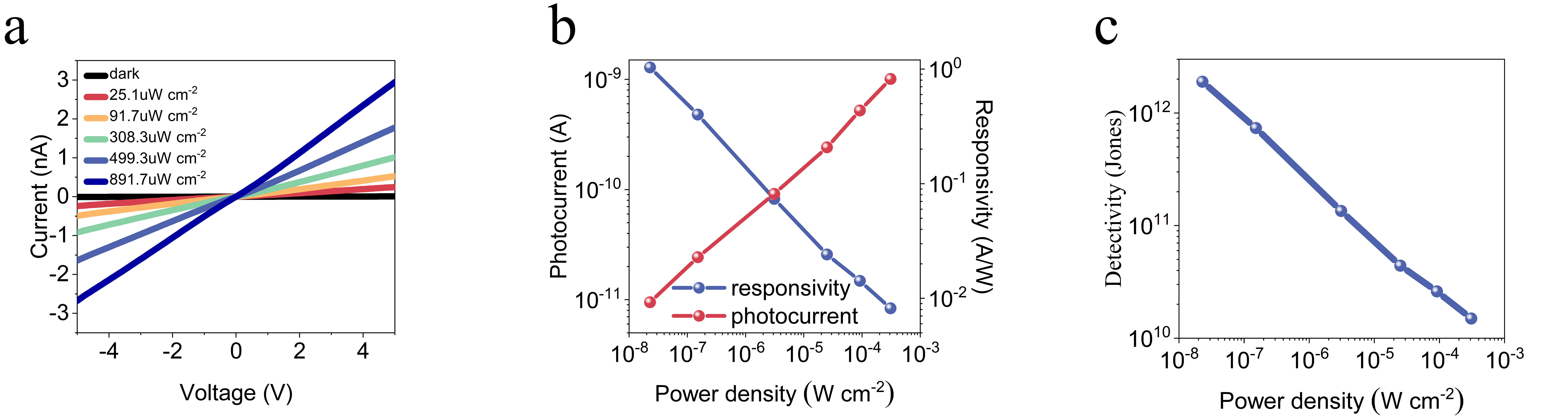


**Figure S5.** Characterization of flexible MAPbI_3_ NA photodetector: (a) I-V curve of the photodetector in the dark state and under 365 nm light irradiation; (b) the photogenerated current and the corresponding responsivity of the device under different light intensity 365 nm light irradiation at a bias voltage of 5 V; (c) the dependence of the detectivity of the device on the intensity, at a bias voltage of 5 V, insert: noise power density spectra of the device at 5 V bias.


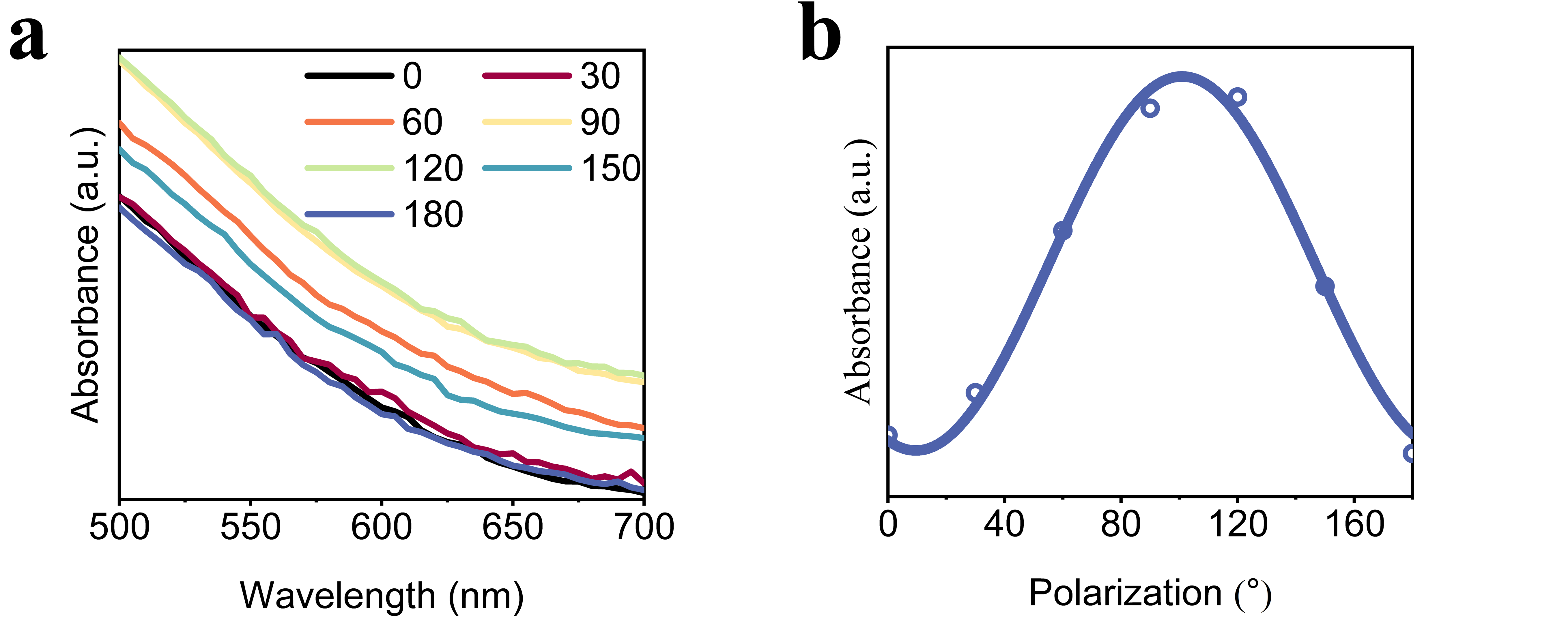


**Figure S6.** Polarization characterization of flexible MAPbI_3_ NA: (a) absorption spectra of flexible MAPbI_3_ NA for polarized light at various angles; (b) the relationship between the absorption coefficient of 600 nm light and the polarization angle.


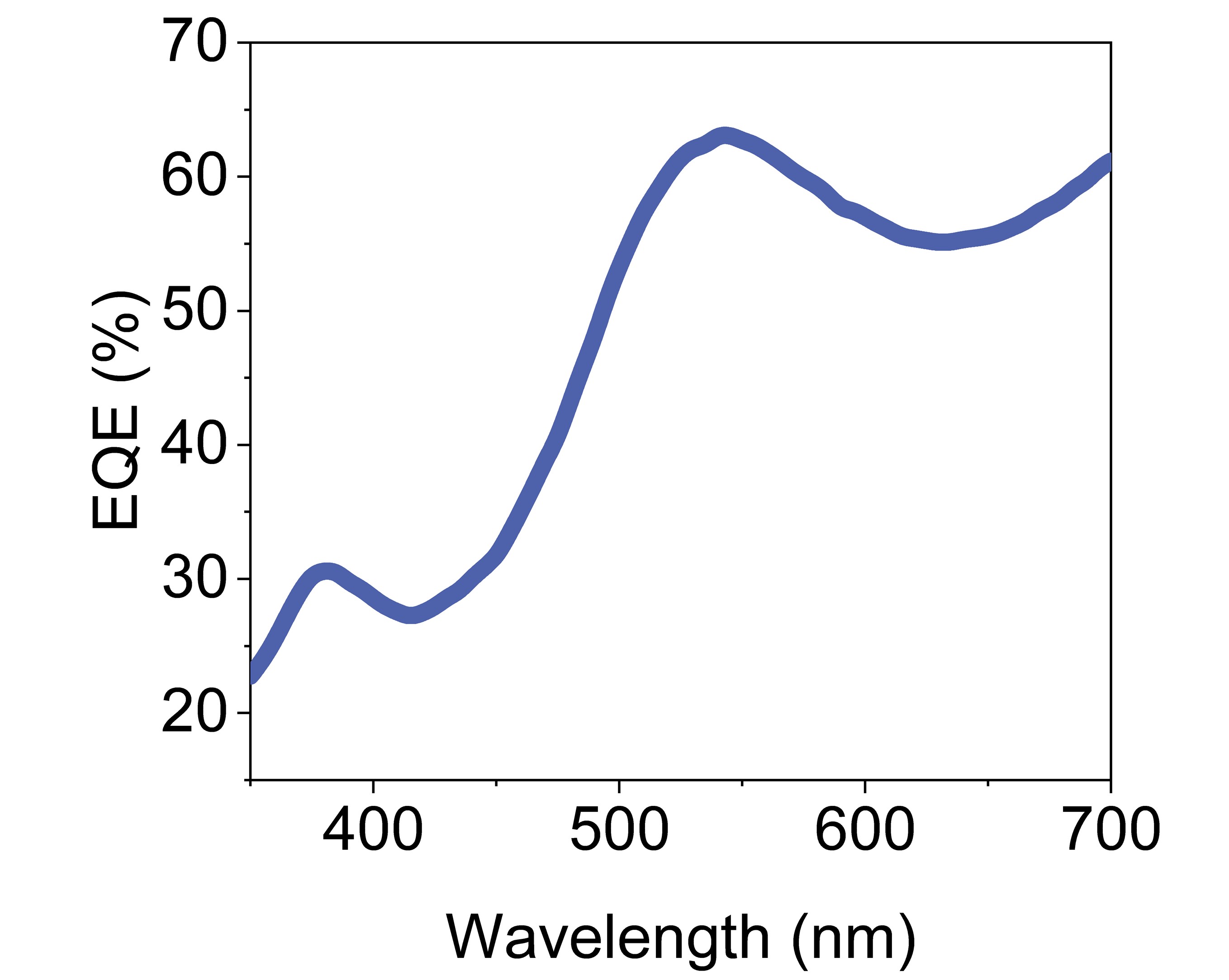


**Figure S7**. External quantum efficiency curve of commercial silicon detectors.


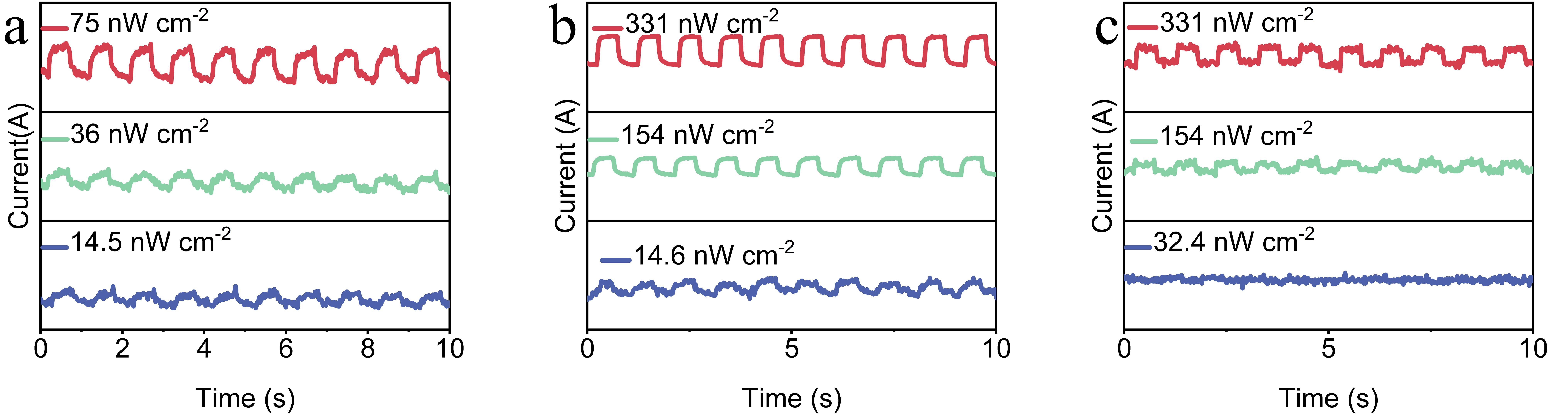


**Figure S8.** Weak light detection performance of MAPbI_3_ NA photodetector:(a) MAPbI_3_ NA photodetector light response at 365 nm; (b) MAPbI_3_ NA photodetector light response at 660 nm; (c) commercial silicon detector light response at 660 nm.


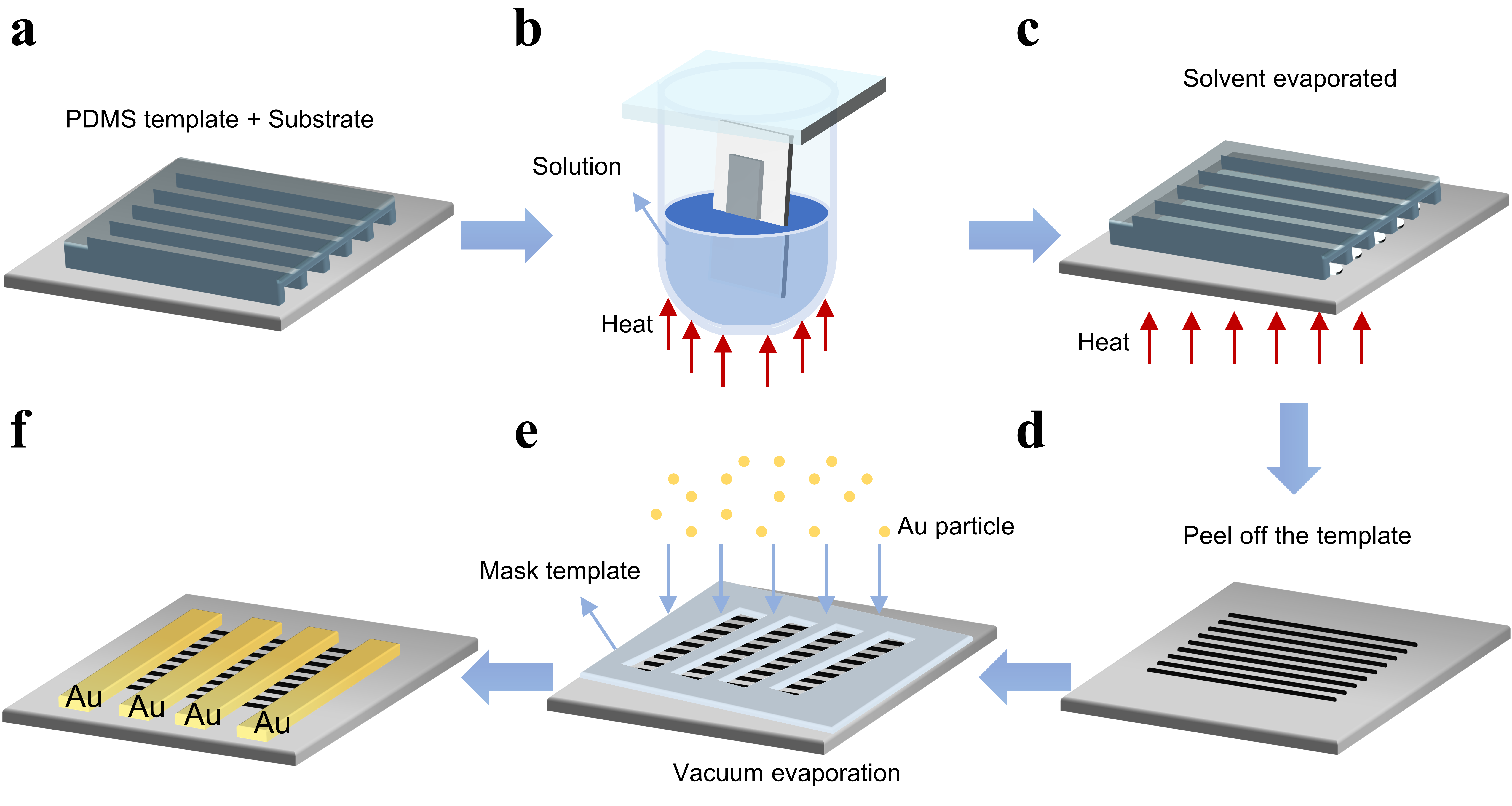


**Figure S9.** The fabrication process for the nanowire array photodetector: (a) the PDMS template with nanostructure is fitted to the substrate to form periodic capillary; (b) the substrate is enclosed within a closed container containing perovskite precursor solution and subjected to thermal treatment; (c) the substrate is extracted from the container and transferred onto a heated table set at a specific temperature to conduct an annealing process; (d) peel off the PDMS template, the nanowires are arranged periodically on the substrate; (e) vacuum deposition of Au electrodes, with the shape of the metal electrodes controlled by a mask template; (f) schematic diagram of a nanowire array photodetector.

**References**:

1. Chen, Y.; Zhang, J.; Zhou, J.; Chu, Y.; Zhou, B.; Wu, X.; Huang, J., Long-Term Stable and Tunable High-Performance Photodetectors Based on Perovskite Microwires. *Adv. Optical Mater.* ***2018****, 6,* 1800469.

2. Gao, L.; Zeng, K.; Guo, J.; Ge, C.; Du, J.; Zhao, Y.; Chen, C.; Deng, H.; He, Y.; Song, H.; Niu, G.; Tang, J., Passivated Single-Crystalline CH_3_NH_3_PbI_3_ Nanowire Photodetector with High Detectivity and Polarization Sensitivity. *Nano Lett*. ***2016****, 16,* 7446-7454.

3. Li, S.; Li, Y.; Shi, Z.; Lei, L.; Ji, H.; Wu, D.; Xu, T.; Li, X.; Du, G., Fabrication of Morphology-Controlled and Highly-Crystallized Perovskite Microwires for Long-Term Stable Photodetectors. *Sol. Energy Mater. Sol. Cells* ***2019****, 191,* 275-282.

4. Wu, D.; Zhou, H.; Song, Z.; Zheng, M.; Liu, R.; Pan, X.; Wan, H.; Zhang, J.; Wang, H.; Li, X.; Zeng, H., Welding Perovskite Nanowires for Stable, Sensitive, Flexible Photodetectors. *ACS Nano* ***2020****, 14,* 2777-2787

5. Huang, R.; Lin, D.-H.; Liu, J.-Y.; Wu, C.-Y.; Wu, D.; Luo, L.-B., Nanochannel-Confined Growth of Crystallographically Orientated Perovskite Nanowire Arrays for Polarization-Sensitive Photodetector Application. *Sci. China Mater.* ***2021,*** *64,* 2497-2506.

6. Deng, W.; Zhang, X.; Huang, L.; Xu, X.; Wang, L.; Wang, J.; Shang, Q.; Lee, S.-T.; Jie, J., Aligned Single-Crystalline Perovskite Microwire Arrays for High-Performance Flexible Image Sensors with Long-Term Stability. *Adv. Mater.* ***2016,*** *28,* 2201-2208.

7. Liu, Y.; Li, F.; Veeramalai, C. P.; Chen, W.; Guo, T.; Wu, C.; Kim, T. W., Inkjet-Printed Photodetector Arrays Based on Hybrid Perovskite CH_3_NH_3_PbI_3_ Microwires. *ACS Appl. Mater. Interfaces* ***2017****, 9,* 11662-11668.

8. Li, J.; Liu, Y.; Ren, X.; Yang, Z.; Li, R.; Su, H.; Yang, X.; Xu, J.; Xu, H.; Hu, J.-Y.; Amassian, A.; Zhao, K.; Liu, S. (F.), Solution Coating of Superior Large-Area Flexible Perovskite Thin Films with Controlled Crystal Packing. *Adv. Optical Mater*. ***2017****, 5,* 1700102.

9. Wu, D.; Xu, Y.; Zhou, H.; Feng, X.; Zhang, J.; Pan, X.; Gao, Z.; Wang, R.; Ma, G.; Tao, L.; Wang, H.; Duan, J.; Wan, H.; Zhang, J.; Shen, L.; Wang, H.; Zhai, T., Ultrasensitive, flexible perovskite nanowire photodetectors with long-term stability exceeding 5000 h. *InfoMat* ***2022****, 4,* e12320.
